# Thyroid nodules ≤ 1 cm and papillary thyroid microcarcinomas: Brazilian experts opinion

**DOI:** 10.20945/2359-3997000000166

**Published:** 2019-08-28

**Authors:** Pedro Weslley Rosario, Laura Sterian Ward, Hans Graf, Fernanda Vaisman, Gabriela Franco Mourão, Mario Vaisman

**Affiliations:** 1 Santa Casa de Belo Horizonte Minas Gerais MG Brasil Santa Casa de Belo Horizonte, Minas Gerais, MG, Brasil; 2 Universidade Estadual de Campinas Faculdade de Ciências Médicas Universidade Estadual de Campinas São Paulo SP Brasil Faculdade de Ciências Médicas da Universidade Estadual de Campinas (Unicamp), São Paulo, SP, Brasil; 3 Universidade Federal do Paraná Universidade Federal do Paraná Curitiba PR Brasil Universidade Federal do Paraná (UFPR), Curitiba, PR, Brasil; 4 Universidade Federal do Rio de Janeiro Universidade Federal do Rio de Janeiro Rio de Janeiro RJ Brasil Universidade Federal do Rio de Janeiro (UFRJ), Rio de Janeiro, RJ, Brasil; 5 Instituto Nacional do Câncer Instituto Nacional do Câncer Rio de Janeiro RJ Brasil Instituto Nacional do Câncer (INCA), Rio de Janeiro, RJ, Brasil

**Keywords:** Fine needle aspiration, thyroid papillary microcarcinoma, active surveillance, lobectomy

## Abstract

The indolent evolution of low-risk papillary thyroid microcarcinoma (mPTC) in adult patients and the consequences of thyroidectomy require a revision of the management traditionally recommended. Aiming to spare patients unnecessary procedures and therapies and to optimize the health system in Brazil, we suggest some measures. Fine-needle aspiration of nodules ≤ 1 cm without extrathyroidal extension on ultrasonography should be performed only in nodules classified as “very suspicious” (i.e., high suspicion according to ATA, high risk according to AACE, TI-RADS 5) and in selected cases [age < 40 years, nodule adjacent to the trachea or recurrent laryngeal nerve (RLN), multiple suspicious nodules, presence of hypercalcitoninemia or suspicious lymph nodes]. Active surveillance (AS) rather than immediate surgery should be considered in adult patients with low-risk mPTC. Lobectomy is the best option in patients with unifocal low-risk mPTC who are not candidates for AS because of age, proximity of the tumor to the trachea or RLN, or because they opted for surgery. The same applies to patients who started AS but had a subsequent surgical indication not due to a suspicion of tumor extension beyond the gland or multicentricity. Molecular tests are not necessary to choose between AS and surgery or, in the latter case, between lobectomy and total thyroidectomy. The presence of RAS or other RAS-like mutations or BRAFV600E or other BRAF V600E-like mutations should not modify the management cited above; however, the rare cases of mPTC exhibiting high-risk mutations, like in the TERT promoter or p53, are not candidates for AS.

## INTRODUCTION

The Brazilian National Institute of Cancer (INCA) estimates an incidence of 1,570 new thyroid tumors in males and 8,040 in females for each year of the 2018-2019 biennium, with an estimated risk of 1.49 and 7.57 cases per 100,000 men and women, respectively (1). As shown in Table 1, thyroid cancer is the fifth most common tumor in women.

**Table 1:** INCA estimates of tumors in Brazilian women for 2018-2019

Site	Number of cases	%
Breast	59,700	29.5%
Colon and rectum	18,980	9.4%
Uterine cervix	16,370	8.1%
Trachea, bronchus and lung	12,530	6.2%
Thyroid	8,040	4%
Stomach	7,750	3.8%
Uterine body	6,600	3.3%
Ovary	6,150	3%
Central nervous system	5,510	2.7%
Leukemias	4,860	2.4%

INCA: *Instituto Nacional de Câncer* (National Cancer Institute).

The incidence of thyroid cancer varies considerably across the different regions of Brazil (1). The incidence is 9.75 cases/100,000 women in well-developed regions such as the southeast, but only 2.8/100,000 among women from the poorer northern part of the country (1). The remarkable geographic variation and sex disparity in thyroid cancer rates observed across regions may reflect differences in ascertainment, diagnosis, treatment, and death certification of this disease, as well as healthcare access. The same phenomenon has been observed in other Latina American countries (2). However, it is noteworthy that mortality rates have remained stable or declined in Brazil, as observed in many other countries, suggesting widespread overdiagnosis (detection of tumors that will not cause clinical illness or death) (3,4). Even in a study reporting a divergent result, the increase in mortality/100,000 inhabitants per year was lower than the increase in incidence (5). This finding suggests that, even if some actual increase in thyroid cancer cases exists, overdiagnosis is a predominant factor (5,6). It should also be noted that the slight increase in mortality may in part be iatrogenic - an increase in the number of patients undergoing treatment might lead to a small number of treatment-related deaths, or due to attribution bias - the incorrect assignment of the cause of death to cancer in a person’s medical history rather than correctly to other causes (6).

In addition to the very sensitive ultrasound devices that are largely available and accessible to the general population at relatively low cost, new detection techniques now routinely reveal small tumors. These diagnoses may cause anxiety not only in the patient and his family, but also in the physician. The easiest and most practical way to deal with the situation is often immediate surgery. In the case of thyroid tumors, the procedure is often followed by unnecessary and even harmful radioiodine administration. The burden of such interventions has dramatically increased in the past years.

Recent data from South Korea suggest that the epidemic of thyroid tumors observed in that country dramatically dropped to before ultrasound screening levels after a group of physicians, supported by the media, led a campaign against routine screening for thyroid cancer (7,8). In fact, the United States Preventive Services Task Force (USPSTF) (9) and the American Thyroid Association (10) advocate against screening for thyroid cancer in asymptomatic adults.

There is an urgent need for a new appraisal of the management of thyroid nodules and tumors. In addition, it is very important to make it clear to patients that immediate interventions and aggressive procedures do not necessarily promote better outcomes and may even not be the best choices.

## PAPILLARY THYROID MICROCARCINOMAS

Papillary microcarcinomas are frequently detected during examination of the thyroid in patients who died of unrelated causes and without a history of thyroid malignancy or in patients submitted to thyroidectomy due to benign disease of the gland. This frequency ranges from 1% to 8% in Brazil (11-14). Moreover, the prevalence of papillary thyroid carcinoma (PTC) detected by ultrasonographic screening in subjects without a family history of thyroid cancer or a history of radiation exposure, who had no abnormalities upon thyroid palpation, was reported to be 1.2% (15). As observed around the world, an enormous disproportion exists in Brazil between this frequency of “occult” PTC and the number of patients diagnosed with this tumor (1,16-18). Since many of the last cases are currently discovered “incidentally” during imaging examination of asymptomatic individuals without palpable thyroid nodules, this disproportion is even higher if we consider only clinically apparent tumors. This great disparity *per se* shows that the progression of PTC to a clinically manifest stage occurs only in a small portion of these tumors.

As a confirmation, several studies evaluating the low-risk papillary thyroid microcarcinomas (mPTC) in adults (19-23) have shown that tumor growth is uncommon and that the development of apparent lymph node metastases is even rarer. So far there are no reports of the occurrence of distant metastases (Table 2). Curiously, even a reduction of mPTC has been observed in 5% to 15% of patients (20,21,24,25). Regarding the characteristics at the time of diagnosis, patient age has been related to the risk of tumor growth, which is higher in young individuals (19-21,23). One study also associated serum TSH > 2.5 mIU/L with a higher risk of tumor growth (26). Importantly, low-risk mPTC is defined in the absence of known distant metastases, apparent extrathyroidal invasion or lymph node involvement, when the tumor is not adjacent to the trachea or recurrent laryngeal nerve, and when fine-needle aspiration (FNA) is not suggestive of an aggressive subtype.

**Table 2:** Natural history of low-risk papillary thyroid microcarcinoma in adult patients

Study (country)	Number of patients	Time of follow-up	Outcomes
Ito and cols., 2014 (Japan) (19)	1,235	18-227 months (mean 60)	Growth: 4.6% LNM: 1.5% Distant metastases: 0
Sugitani 2018 (Japan) (20)	426 (532 lesions)	1-26 years (mean 8.7)	Growth: 8.4% LNM: 0.9% Distant metastases: 0
Sugitani 2018 (Japan) (20)	101	1-5 years (mean 2.4)	Growth: 6% LNM: 1% Distant metastases: 0
Tuttle and cols., 2017^†^ (United States) (21)	291	6-166 months (median 25)	Growth: 3.8% LNM: 0 Distant metastases: 0
Sanabria 2018^†^ (Colombia) (22)	57	0-54 months (median 13)	Growth: 3.5% LNM: 0 Distant metastases: 0
Oh and cols., 2018 (South Korea) (23)	370	21-47 months ^§^ (median 32)	Growth: 3.5% LNM: 1.3% Distant metastases: 0
-	2,480	-	Growth: 5.2% LNM: 1.1% Distant metastases: 0

LNM: lymph node metastases. Growth = enlargement ≥ 3 mm.

^†^ These studies included patients with tumors measuring 1 to 1.5 cm. ^§^ Interquartile.

Even without the adverse effects of radioactive iodine and the risks of exogenous suppression of TSH, therapies no longer recommended for low-risk mPTC (27) but still prescribed for many patients with this tumor, the undesirable consequences of surgery remain. Although uncommon, hypoparathyroidism and recurrent laryngeal nerve injury can occur even when thyroidectomy is performed by experienced surgeons. Inevitable after total thyroidectomy, levothyroxine replacement therapy is also commonly required in patients with PTC after a lobectomy (28,29). The difficulty of achieving sustained control, patient dissatisfaction and compromised quality of life during this lifelong therapy have been shown in a Brazilian multicenter study (30,31).

## RECOMMENDATIONS

The low risk of progression and the consequences of surgery, even lobectomy performed by experienced surgeons, are factors that lead to the rethinking of immediate surgery as the best option in patients with low-risk mPTC and that have resulted in the strong current trend of considering active surveillance as the most adequate management in this situation. In clinical practice, three approaches have been adopted to avoid overtreatment of these patients.

First, FNA of nodules ≤ 1 cm without apparent extrathyroidal invasion or lymph node disease on ultrasonography (US), which was already restricted to “very suspicious” nodules, is now only recommended in selected cases or is no longer indicated even in these nodules (Table 3).

**Table 3:** Recommendation of fine-needle aspiration (FNA) in Europe and the United States for adults with “very suspicious”§ nodules ≤ 1 cm without extrathyroidal extension or lymph node involvement on ultrasonography

Reference	Recommendation
ATA 2016 (10)	Does not recommend FNA.
AACE 2016 (32)	In nodules with a diameter 5-10 mm consider either FNA sampling or watchful waiting on the basis of the clinical setting and patient preference. Specifically, FNA is recommended for the following nodules: subcapsular or paratracheal lesions, positive personal or family history of thyroid cancer.
Leboulleux and cols., 2016 (33)	FNA if family history of thyroid cancer or previous head and neck external beam radiation, or suspicion of one or more of the following: microcarcinoma adjacent to recurrent laryngeal nerve or to trachea, multinodular thyroid.
ETA 2017 (34)	Does not recommend FNA.
ACR 2017 (35)	Biopsy of 5- to 9-mm TI-RADS 5 nodules may be appropriate under certain circumstances. The determination to perform FNA will involve shared decision making between the referring physician and the patient.
NCCN 2019 (36)	Does not recommend FNA.
Italian Societies 2018 (37)	Diameter 5-9 mm: either FNA sampling or US monitoring on the basis of clinical setting and patient preference. FNA is recommended for subcapsular, posterior or paratracheal lesions, or in case of clinical thyroid cancer risk factors.

^§^ “High suspicion” (10), Class 3 (“high risk”) (32), EU-TIRADS 5 (34), or ACR TI-RADS 5 (35).

ATA: American Thyroid Association; AACE: American Association of Clinical Endocrinologists; ETA: European Thyroid Association; ACR: American College of Radiology; NCCN: National Comprehensive Cancer Network.

*Recommendation 1*: This panel recommends FNA for nodules ≤ 1 cm if they are “very suspicious” on US and, additionally, if any of the features listed in Table 4 is observed. Obviously, patients initially not submitted to FNA should be monitored and FNA may be indicated during follow-up (Table 4).

**Table 4:** Fine-needle aspiration (FNA) recommended by this panel for adults with “very suspicious”§ nodules ≤ 1 cm on ultrasonography (US) and follow-up of patients not submitted to FNA

**Indication for initial FNA:** Age < 40 years or known distant metastases Nodule with extrathyroidal extension on US, adjacent to the trachea or recurrent laryngeal nerve, or multiple “suspicious” nodules Presence of suspicious lymph nodes^§§^ Hypercalcitoninemia suggestive of medullary thyroid carcinoma (if calcitonin is obtained) In cases in which confirmation by FNA that the nodule is a papillary microcarcinoma would lead to the indication of therapy with levothyroxine (see Table 5) Patient’s desire
**Follow-up of patients initially not submitted to FNA:** Clinical examination Ultrasonography (US) aimed at: (i) dimensions and volume of the nodule, (ii) emergence of new “suspicious” nodule(s), (iii) extrathyroidal invasion, (iv) relationship of the nodule with the trachea and recurrent laryngeal nerve, and (v) suspicious lymph node(s) Serum TSH Periodicity: US every 6 months in the first 12 months and then annually in the absence of growth FNA if the nodule monitored exceeds 1 cm, exhibits rapid growth (100% increase in volume in less than 2 years), or extrathyroidal extension; suspicious lymph node on US; new data that would indicate initial FNA

^§^ “High suspicion” (10), Class 3 (“high risk”) (32), EU-TIRADS 5 (34), or ACR TI-RADS 5 (35).

^§§^ Combined with FNA of the lymph node (cytology and thyroglobulin measurement in its washout).

*Recommendation 2*: In the case of patients submitted to FNA whose result of cytology or molecular testing is compatible with PTC, this panel recommends active surveillance rather than immediate surgery as an option in many cases (Table 5). It is also important to note here that patients initially not submitted to surgery should be followed up closely and thyroidectomy might be indicated later (Table 5).

**Table 5:** Criteria suggested by this panel for the choice of active surveillance over immediate surgery in adult patients with papillary thyroid microcarcinoma and follow-up of patients not submitted to surgery

Clinical criteria: age > 40 years, absence of known distant metastases, and clear agreement of the patient or legal representative Ultrasonographic criteria: single tumor, not adjacent to the trachea or recurrent laryngeal nerve, and absence of apparent extrathyroidal invasion or lymph node involvement FNA criteria: cytology or molecular test (if obtained) not suggestive of aggressive subtype Laboratory criteria: absence of hypercalcitoninemia suggestive of medullary carcinoma (if calcitonin is obtained)
Follow-up of patients with low-risk papillary thyroid microcarcinoma not submitted to surgery: Clinical examination Ultrasonography (US) aimed at: (i) dimensions and volume of the tumor, (ii) emergence of new “suspicious” nodule(s), (iii) extrathyroidal invasion, (iv) relationship of the tumor with the trachea and recurrent laryngeal nerve, and (v) suspicious lymph node(s) Serum TSH.^†^ Periodicity: US every 6 months in the first 12 months and then annually in the absence of progression Surgery if the patient no longer meets the above criteria for active surveillance, continues with these criteria but exhibits rapid tumor growth (100% increase in volume in less than 2 years), patient’s desire

^†^ Therapy with L-T4 to maintain TSH between 0.5 and 2 mIU/L may be prescribed for patients with TSH between 2.5 and 4 mIU/L less than 65 years and is recommended for patients with TSH > 4 mIU/L.

One noteworthy finding is the excellent evolution of patients whose surgery was only performed later for several reasons, including tumor progression. Among 303 patients who underwent surgery after a period of active surveillance, highlighting that most of them did not receive radioactive iodine, only one case of short-term local recurrence (0.35%) was observed (19-21,23), a rate similar to that found in patients operated on immediately after diagnosis.

*Recommendation 3*: This panel recommends lobectomy as the best option for patients with mPTC who are not candidates for active surveillance because of age, proximity of the tumor to the trachea or recurrent laryngeal nerve, or because they opted for surgery, but who meet the remaining criteria reported in Table 5. The same applies to patients who started active surveillance but had a subsequent surgical indication not due to tumor beyond the gland or multicentricity.

Recently, treatment of mPTC with ablative techniques such as laser, radiofrequency, microwave or percutaneous ethanol injection has been reported (38-41). Although studies have demonstrated the short-term efficacy and safety of these procedures, it is the current opinion of this panel that there is no evidence of their superiority over active surveillance in patients who are candidates for the latter, or of their superiority over surgery when it is indicated and feasible.

*Recommendation 4:* This panel considers that, provided the criteria of Tables 5 and 6 are met, molecular tests are not necessary to choose between active surveillance and surgery or, in the latter case, between lobectomy and total thyroidectomy. In cases in which molecular tests are obtained, the presence of *RAS* or other *RAS*-like mutations (e.g., *PAX8/PPARG* rearrangement) or *BRAFV600E* or other *BRAF V600E*-like mutations (e.g., *RET/PTC* fusions) should not modify the management cited above. Mutations in the *TERT* promoter were recently reported to correlate strongly with aggressiveness in advanced forms of thyroid cancer. Although they are uncommon in mPTC apparently restricted to the thyroid (43) and have not been associated with the growth or development of lymph node metastases in mPTC under active surveillance (44), this panel currently considers that the rare cases of mPTC exhibiting high-risk mutations, like in the *TERT* promoter or p53, are not candidates for active surveillance.

**Table 6:** Criteria suggested by this panel for the choice of lobectomy in patients with papillary microcarcinoma and for the indication of surgical complementation in the short-term.

Patients who are not candidates for active surveillance due to age, proximity of the tumor to the trachea or recurrent laryngeal nerve, and preference for surgery, but who meet the remaining criteria of Table 5; or patients who had a surgical indication during active surveillance not due to tumor beyond the gland or multicentricity Patient accepting possible complementation and not exhibiting high anesthetic/surgical risk Contralateral lobe without a very suspicious nodule or nodule > 1 cm or multiple nodules Absence of apparent extrathyroidal invasion and lymph node involvement during perioperative evaluation
Unnecessary criteria but that reinforce the choice of lobectomy: Euthyroidism with preoperative TSH ≤ 2 mIU/L and without Hashimoto’s thyroiditis Negative anti-Tg antibody Absence of nodule in the contralateral lobe
If accepted and feasible, complementation of lobectomy in the short-term if: Stage T3b, vascular invasion, aggressive histological subtype, compromised margins Postoperative ultrasonography demonstrating lymph node metastases not detected pre- or perioperative (rare) Unstimulated Tg > 30 ng/mL (10,42)^§^ a few months after surgery and with controlled TSH (rare)

^§^ It can be half the upper limit of the normal range obtained for healthy individuals.
